# On the Pathology and Treatment of Sanguineous Pelvic Cysts

**Published:** 1853-02

**Authors:** 


*On the Pathology and Treatment of Sanguineous Pelvic Cysts.*—Dr. Tilt read a paper on a variety of pelvic tumors, on which the French pathologists have during the last year thrown great light, the cyst being in such cases formed by a considerable quantity of blood effused in the pelvic portion of the peritoneum, or externally to the peritoneum, so as to constitute extra-peritoneal or intra-peritoneal cysts. Dr. Tilt first related several cases from his own practice, from that of Dr. Bell of Glasgow, and from that of Dr. H. Bennet; by which it appeared that the disease was preceded by menstrual irregularities, and that its origin generally coincided with the suppression or the non-appearance of the catamenia; after which ensued hypogastric swelling, then local pelvic peritonitis, on the subsidence of which a globular tumor was found to fill, more or less, the pelvis, interfering with the passage of both fæces and urine. Dr. Tilt then elucidated the pathology of the previous cases by several others which had terminated fatally in the Paris hospitals, and where the post-mortem examinations were exhibited to the Société de Chirurgie of Paris. The causes of extra-peritoneal sanguineous cysts were extremely obscure. In a case related in "Guy's Hospital Reports," the effusion of blood was caused by the rupture of an aneurismal sac. In another, occurring in the practice of the celebrated Margolin, it was caused by the rupture of varicose sub-peritoneal veins. Light had been thrown on the causes of the intra-peritoneal variety by the post-mortem appearance of a patient under the care of Denonvilliers, of the Hôpital Ste. Marguerite, in Paris. The blood was found to have come from several small ovarian cavities, some of which still contained blood-clots; and in discussing the case, Lenoir, Nelaton and other surgeons of eminence, admitted that in this and similar cases the pelvic tumor was formed by an ovarian hemorrhage taking place during the process of ovulation. Dr. Tilt admitted the explanation, and gave it strength by reminding the Society that during the process of ovulation the ovaries greatly increased in size; that in the normal state the blood-clot taking the place of the ovum was about the size of a cherry; that microscopical observers had sometimes found the capillary vessels much enlarged and broken in the vicinity of the vesicle, and the ovarian tissue so softened that it would tear on the slightest touch, and let the blood-clot escape. Dr. Tilt gave a still surer footing to the views of the French pathologists on this point, by relating a case which had lately occurred in the Lyons hospital, where, after flooding at three successive menstrual periods, peritonitis supervened, and on opening the body, a blood-clot of the size of a horse-bean was found protruding between the rent lips of the ovarian follicle. Having thus established the pathology of intra-peritoneal sanguineous cysts, Dr. Tilt proceeded to illustrate their history by other interesting cases which had lately occurred in the Paris hospitals. One, for instance, was mistaken by Malgaigne for a fibrous uterine tumor. He slit up the neck of the womb to enucleate the supposed tumor, but, finding his mistake, he punctured it, and gave issue to a large quantity of syrupy blood. Hemorrhage from the wounded artery of the neck of the womb could not be controlled, and the patient died. Another case was mistaken for ovarian dropsy, others for metritis. Dr. Tilt observed that sanguineous pelvic cysts did not usually terminate fatally, but often by resolution; the lining membrane of the cyst absorbing its contents, which thus became thicker and thicker, and the tumor diminished. Sometimes, however, the contents were evacuated by rupture of the cyst per rectum or per vaginam. Dr. Tilt recommended the cysts to be left to themselves when small, and to increase absorption by moderate venesection, purgatives and low diet; but if they attained a considerable volume, so as to interfere much with micturition and defæ-

cation, he advised the tumor to be punctured per vaginam with a long trocar. 1st. Because this plan presents a better chance of avoiding wounding the arteries. 2d. Because the trocar being left in the wound permits the gradual evacuation of the blood. 3d. Because it allows the possibility of making injections. But Dr. Tilt likewise admitted the necessity of widely opening the tumor should it contain large fibrinous clots, or if it gave rise to a fetid discharge. With regard to the frequency of the disease, Dr. Tilt did not consider it a common occurrence; and although some twenty cases had been published during the past year by eminent French surgeons or obstetric practitioners, it was so little known in England, that several talented physician-accoucheurs had assured the author that they had not met with it. Dr. Tilt, therefore, submitted, that they might have been mistaken for other complaints; for metritis and incipient pelvic abscess, when the collection of blood is small; for an ovarian tumor when the collection is very considerable; and sometimes for pelvic abscesses, as in Dr. Bell's case, when the blood-clots were mixed with pus. He, therefore, believed that similar cases might be found recorded in English journals by those who would have time and patience to hunt for them.

Dr. De Meric inquired if Dr. Tilt had noticed pulsation in the tumors which he had described. In the description of one of the cases, at least, he had spoken of enlargement of the vessels, and of fibrinous deposit, &c.; might not the tumor in this instance have been aneurismal? In those cases in which recovery had taken place, which was the pathological process by which this was effected? The author had stated that he considered that hemorrhage had occurred from the ovary in these cases at some time or other during menstruation. Now this was no doubt from excitement of the ovary. What, then, was the cause of the hemorrhage? Had coïtus anything to do with it, or any other stimulant? If we could trace the hemorrhage to any such cause, we might warn the patient. There were, no doubt, however, other circumstances connected with the menstrual period which were favorable to the development of these tumors.

Mr. Brown regretted that so little of the paper allowed of discussion, as only one of the cases had fallen under the observation of Dr. Tilt himself. He (Mr. Brown) was inclined to think that one, if not two, of the cases, was an iliac aneurism. In the case related as occurring in Dr. Tilt's own practice, no doubt the use of the trocar was proper; but he (Mr. Brown) did not think it would be so in the other cases. He did not agree with Dr. Tilt that these tumors were likely to be mistaken for uterine enlargements, as the uterus could be distinctly felt on examination; but he had seen instances in which they might be mistaken for ovarian tumors. In one case he recollected a sanguineous swelling near to the uterus, which burst into the rectum, and had been mistaken for an ovarian tumor. When the nature of the tumor could be determined by the history, and the mischief was localized, the plan of treatment recommended in the paper was proper, but it should not be resorted to when the swelling came on suddenly. Then a very different mode of proceeding was necessary. He regarded these cases generally as a sort of uterine apoplexy from obstructed menstruation, and situated in the cellular tissue. He hoped at a future time Dr. Tilt would bring forward cases which would fall under his own observation, as some, no doubt, soon would.

Dr. SAMUEL GRIFFITH found the same difficulty as the last speaker in discussing the paper; but he would briefly relate two cases which had fallen under his own notice, and which appeared to be somewhat analogous to the one detailed by Dr. Tilt. The first was the case of a woman who had been ailing for some time, and had pain and hardness in one of the ovaries, with occasional shivering, and a mucous discharge from the vagina, unaccompanied by ulceration of the cervix uteri. She suddenly became worse, and symptoms of acute peritonitis set in without any apparent cause. Treatment was unavailing, and she died on the seventh day. On examination, it was found that profuse hemorrhage had taken place into the cavity of the peritoneum. It was not confined to the pelvis, and had given rise to suppuration. The point from whence the hemorrhage proceeded could not be detected, and he could not therefore assert that it was from the ovary. In such a case none but general treatment could be adopted, as there were no symptoms to indicate from what cause the disease arose. In another case, which was under treatment in St. Thomas's hospital, the subject was an over-worked servant-girl, somewhat out of health from suppressed menstruation. She had a tumor in the lower part of the abdomen, which was swollen as large as in the seventh month of pregnancy. It resembled pregnancy also in shape. It was painful and tender on pressure; the os and neck of the uterus were healthy. At the end of two months the tumor suppurated, and opened externally in the lower part of the abdomen. The tumor, however, remained of the same size and of the same stony hardness. There was no vaginal discharge. This case, at its termination, might be found to bear some analogy to the cases detailed in Dr. Tilt's paper.

Mr. DENDY regarded Dr. Tilt's cases as mere effusions of blood into the cellular membrane, round which a cyst had been formed, and a tumor was the result. Cases somewhat analogous were occasionally observed during parturition, from effusion of blood into the cellular membrane of the vagina.

Dr. CHOWNE would not enter into the discussion, as he had not seen such cases as those detailed, which were very rare; and if Dr. Tilt waited until he could speak largely from his own experience upon the matter, as was suggested by Mr. Brown, it was probable he would long remain silent. He regarded the cases related as differing in their character, but that generally these blood-tumors were the result of effusion of that fluid into the cellular membrane. He found fault somewhat with the title of the paper, which, strictly speaking, was not on "*pelvic* sanguineous tumors."

Dr. TILT, in reply to Dr. De Méric's suggestion, that some of the cases described might have been cases of aneurism of some large abdominal vessel, observed, that with Dr. Lever's patient he had stated that this was the case, and it might therefore be met with again as a cause of extra-peritoneal sanguineous tumors; but that the other seven cases related could not be confounded with aneurism, for in three out of the seven there were post-mortem examinations, and the other four were related by accomplished observers, able to detect a pulsating tumor, of chronic growth, from those described in the paper. Dr. De Méric also asked whether ovarian inflammation did not play a part in this disease; but Dr. Tilt thought that care should be taken in ascribing every morbid action to one pathological condition, and said that, when taking into consideration the action of the ovaries, we must not think of these organs as they are met with in the dead body, but as seen by Verdier and Dr. Oldham, when accidentally placed outside the inguinal canal, in which case, at the menstrual periods, they have been observed to swell *enormously*; that we must think of the softened state of the ovarian tissue surrounding the rent follicle of the ruptured capillaries, attested by microscopical observers, and also of the ruptured blood-vessels seen by them. Dr. Tilt believed, with the French authorities already quoted, that in these cases of *intra-peritoneal* sanguineous cysts, the blood trickled from the rent capillaire, and falling into the recto-peritoneal pouch, caused peritonitis, and therefore a cyst containing blood. Such were Dr. Tilt's views of the pathology of such cases. He could not understand how obstructed circulation could account for them, as had been suggested by Mr. Dendy; he did not admit that the blood in such cases was merely effused in the areolar tissue, for on post-mortem examinations it was proved that the blood had broken down the areolar tissue, displacing it and the pelvic organs so as to constitute a cyst. Neither could he accept Mr. Brown's explanation, that such sanguine collections were formed by the blood permeating the outward surface of the womb, because, whenever death had afforded an opportunity of investigating the case, a less problematical explanation had been found. Dr. Griffith did not see how fluctuation could be felt if the tumor contained blood, but Dr. Tilt reminded him that in some of the cases, the blood, like menstrual fluid in a distended womb, did not coagulate, being a dark syrupy fluid. Fluctuation was of course less marked or imperceptible when the blood was much coagulated, and when its liquid portions were absorbed. To Dr. Chowne's objection, that some of the tumors described could not be fairly called sanguineous, since the contents were purulent as well as sanguineous, Dr. Tilt added that the peritonitis, by which the blood was confined in the pelvic cavity, might pass to the stage of suppuration, and then pus would be voided with the blood. It had been suggested by Mr. Brown that as the cases described were very common, it was a pity Dr. Tilt had not waited so as to build his paper upon his own researches only; but Dr. Tilt replied that his reason for bringing the subject before the notice of the Society was the fact of such cases not having been noticed in England; and he considered it a singular circumstance that Mr. Brown should have frequently met with cases of sanguineous pelvic cysts, when he had been informed by Dr. Murphy and Dr. Oldham, that in their extensive fields of inquiry they had not met with them; that Dr. Chowne and Dr. H. Bennet had but seldom done so; and that they were considered uncommon by many French pathologists who during the past year had drawn attention to the subject.—*Lancet.*

